# Acute Hemorrhagic Pancreatitis

**Published:** 1904-12

**Authors:** L. A. Highland

**Affiliations:** Clinic at the Buffalo Hospital Sisters of Charity


### CLINICAL REPORT.

### Acute Hemorrhagic Pancreatitis.

By L. G. HANLEY, M. D., Buffalo, N. Y.

Reported by L. A. Highland, M. D.
Clinic at the Buffalo Hospital Sisters of Charity.

MRS. M., age 50; family history, negative; personal history, negative, except the usual diseases of childhood. Weight, 150 pounds; height, 5 feet, 4 inches; married twenty years ago, never pregnant; operated on in 1903 for inguinal hernia.

Patient came under my observation May 4, 1904, for gastralgia, with nausea and vomiting. She at this time was subject to severe attacks of gastralgia, coming on from two to three hours after meals. Such attacks were usually relieved by counterirritants and the ordinary household remedies. At this time the patient could retain nothing except tea and coffee. Lavage was instituted and a gastric analysis made, which showed some delayed motility together with the presence of the fermentative acids. Hydrochloric acid was present and starch digestion impaired.

Microscopically the gastric analysis showed a large amount of fat globules and the gross appearance of the contents also suggested fat. A peculiar subjective sensation of the patient at this time was the fact that to her the secretions of the mouth and stomach tasted oily. Lavage was employed every second day. The patient's diet at this time consisted of prepared milk and toast. After a week of lavage her general condition improved and her diet was gradually increased until at the end of two months and a half, she could drink milk and broth, eat scraped beef, toast and cereals. During this treatment of lavage no medicines were given, except a dram of artificial Carlsbad salts in the morning to regulate bowels, and an alkaline gastric sedative. The patient improved to such an extent that all gastralgia had disappeared, no eructations were experienced, headache ceased, and motility of stomach contents was practically normal. She had gained three pounds in weight during the two and a half months of observation. She left the city on a vacation and returned in about four weeks, feeling as well as before and having gained one pound a week.

### PRESENT ILLNESS.

The patient did not consult a physician again until November 3, 1904, at which time she had a very severe attack of gastralgia. Pain was most severe in the epigastric region. At this time she was vomiting food which she had taken at the previous meal. Her temperature was normal; pulse, 70, irregular and of poor tension. She was writhing in pain, and her skin was cold and clammy; half a grain of morphia was administered at 7 p. m. She was put to bed and hot fomentations were applied to the abdomen. Another half grain of morphia was given at 1 a. m. At this time the seat of pain had changed somewhat and was most severe in the region of the right scapula, from whence it radiated down the right arm. Next morning there was marked spasm of the right rectus extending down to the umbilicus. She was vomiting bile, was constipated, pulse, 90; temperature, 99°.

She was seen again in the afternoon of the same day, at which visit her temperature was 99°, and her pulse, 120. A blood count showed 21,000 whites; urinalysis showed granular casts, albumin, but no sugar. A diagnosis of cholecystitis was made and an operation advised. The patient was prepared for operation and an incision made in the right upper quadrant of the abdomen, with the intention of attacking the gallbladder. About one quart of dark fluid flowed from the wound, which resembled blood and bile. When the field of operation was cleared, it was observed that foci of fat necrosis without number, studded the omentum. These varied in size from a millet seed to the size of rice. On opening the gastrocolic omentum, the tissue of the pancreas was found filled with bloodstained fluid. Fat necrosis studded the lesser curvature of stomach.

The gallbladder was drawn into the field of operation, incised and three ounces of dark bile of the consistency of tar flowed out. As far as could be ascertained no calculi were found; neither palpation nor probing the ducts discovered gallstones. A drainage tube was inserted into the gallbladder and the patient was hurriedly removed from operating table.

Eleven days after the operation the patient improved to such an extent that she took nourishment well, the gallbladder still draining, bowels acting well and all indications pointed to complete recovery.

